# Engineering Glutathione Peroxidase-Loaded Polymeric Nanogels Through a Grafting-To Route for Enhanced Enzyme Stability and Activity

**DOI:** 10.3390/polym17233180

**Published:** 2025-11-29

**Authors:** Suman Basak

**Affiliations:** 1 Department of Health Technology, DTU Health Tech, Technical University of Denmark, 2800 Kgs. Lyngby, Denmark; basaksuman8@gmail.com or sumba@kemi.dtu.dk; 2 Department of Chemistry, Technical University of Denmark, 2800 Kgs. Lyngby, Denmark

**Keywords:** glutathione peroxidase nanogels, PEGylated enzyme delivery, epoxy–amine conjugation, antioxidant and ROS Scavenging, controlled RAFT polymerization

## Abstract

Nanogels provide unique opportunities for stabilizing fragile enzymes through soft, hydrated polymer networks. Here, we report the development of a glutathione peroxidase (GPx)-loaded nanogel (GPxNG) engineered via a mild “grafting-to” epoxy–amine coupling strategy to enhance enzyme stability and antioxidant function. An amphiphilic copolymer composed of methacrylated 2,2,6,6-tetramethyl-4-piperidyl (PMA) and glycidyl methacrylate (GMA) was synthesized by controlled reversible addition–fragmentation chain-transfer (RAFT) polymerization using a poly(ethylene glycol) (PEG) macro-chain transfer agent (macro-CTA), yielding well-defined polymer chains with reactive epoxy groups. Covalent conjugation between polymer epoxides and GPx enzyme surface amines generated soft, PEGylated nanogels with high coupling efficiency, uniform particle sizes, and excellent colloidal stability. The engineered nanogels exhibited shear-thinning injectability, robust storage stability, and non-cytotoxic behavior in RAW 264.7 macrophages. Compared with native GPx enzyme, GPxNGs demonstrated significantly enhanced reactive oxygen species (ROS) scavenging activity, including strong inhibition of lipid peroxidation and copper-induced low-density lipoprotein (LDL) oxidation. Importantly, the nanogels preserved GPx enzyme activity after extended storage, freeze–thaw cycles, and repeated catalytic use, whereas the free enzyme rapidly lost function. This protective effect arises from the nanoscale confinement of the GPx enzyme within the flexible PEG-based network, which limits unfolding and aggregation. Overall, this work introduces a simple and biocompatible “grafting-to” nanogel platform capable of stabilizing redox-active enzymes without harsh conditions. The GPx nanogels combine high enzymatic preservation, potent antioxidant activity, and excellent handling properties, highlighting their potential as a therapeutic nanoplatform for mitigating oxidative stress-associated disorders such as atherosclerosis.

## 1. Introduction

Nanogels, a class of nanoscale, crosslinked polymeric networks, have gained significant attention in recent years due to their unique combination of physicochemical tunability, biocompatibility, and aqueous stability [1,2]. Their three-dimensional, hydrophilic structure enables high water retention while maintaining mechanical integrity, allowing them to mimic biological tissues and facilitate efficient diffusion of biomolecules. These properties make nanogels highly attractive for biomedical applications such as drug delivery, enzyme immobilization, and tissue engineering [3,4].

Various synthetic approaches, including emulsion polymerization, self-assembly, and covalent crosslinking, have been developed for nanogel fabrication [5]. Among them, covalent crosslinking methods, particularly grafting-based polymerization strategies, provide superior structural stability and control over size, morphology, and functionality. In this context, the “grafting-to” approach has emerged as an efficient route for enzyme or biomolecule immobilization, offering mild reaction conditions, high coupling efficiency, and preservation of biological activity [6,7].

Nanogels are particularly useful for protecting fragile biomacromolecules such as enzymes, which often suffer from denaturation, aggregation, or loss of activity under physiological or storage conditions [8]. Immobilizing enzymes within a soft polymeric matrix can improve their structural stability, prolong functional lifespan, and allow repeated catalytic use. Additionally, nanogels can be engineered with stimuli-responsive or PEGylated components to enhance solubility, minimize immune recognition, and enable targeted or sustained delivery, key features for therapeutic use [9].

Oxidative stress, resulting from excessive reactive oxygen species (ROS) generation, is implicated in numerous diseases, including cardiovascular disorders, neurodegeneration, and cancer [10]. Among these, atherosclerosis remains a major global health concern characterized by lipid accumulation, inflammation, and oxidative modification of low-density lipoprotein (LDL) [11]. The antioxidant enzyme glutathione GPx enzyme plays a crucial role in neutralizing ROS and protecting cells from oxidative damage by catalyzing the reduction of hydrogen peroxide and lipid peroxides. However, the therapeutic application of GPx enzyme is hindered by its poor stability, short circulation half-life, and susceptibility to proteolytic degradation, underscoring the need for an efficient stabilization and delivery platform [12].

The grafting-to polymerization approach offers a promising strategy to immobilize GPx enzyme within nanogels while retaining its catalytic activity. In this work, we employed an epoxy–amine coupling mechanism to covalently link GPx enzyme onto a reactive amphiphilic copolymer synthesized via controlled RAFT polymerization of methacrylated 2,2,6,6-tetramethyl-4-piperidyl (PMA) and glycidyl methacrylate (GMA) using PEG macro-CTA. The epoxy groups of the polymer readily react with the free amine residues of GPx, forming stable, covalently crosslinked PEGylated nanogels under mild aqueous conditions. This strategy enables precise control over molecular architecture and enzyme loading while avoiding harsh solvents or initiators that could denature the enzyme [13,14,15].

**Figure 1:**
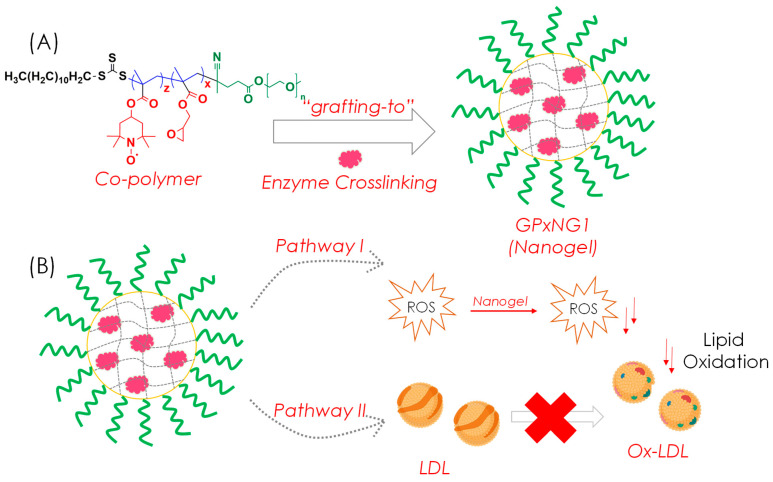
Schematic of the “grafting-to” approach for forming GPx nanogels (GPxNGs): (**A**) covalent conjugation of GPx with the reactive copolymer; (**B**) antioxidant functions of GPxNGs, including ROS scavenging and inhibition of LDL oxidation.

Here, we report the synthesis, characterization, and functional evaluation of PEGylated GPx-conjugated nanogels prepared through a “grafting-to” approach (Figure 1). The nanogels were optimized using a design-of-experiments (DoE) framework to achieve desirable physicochemical properties, including size uniformity, colloidal stability, and biocompatibility. Furthermore, we investigated their antioxidant performance, cytocompatibility, and ability to mitigate LDL oxidation, a key process in atherosclerosis progression. The developed nanogel system demonstrates exceptional enzyme stability, injectability, and ROS-scavenging efficiency, highlighting its potential as a robust antioxidant nanoplatform for oxidative stress-related diseases.

The main objective of this study is to develop a covalently crosslinked PEG-based GPx nanogel using a mild epoxy–amine ‘grafting-to’ strategy that enhances enzyme stability, catalytic durability, and antioxidant performance. Unlike many existing approaches that rely on physical adsorption or non-specific encapsulation, this method enables controlled and stable conjugation under aqueous conditions without harsh reagents. The novelty of this work lies in combining RAFT-derived reactive copolymers with enzyme-friendly conjugation chemistry to achieve high loading efficiency, long-term activity retention, and reusability—features that are often limited in previously reported GPx delivery systems. This platform, therefore, offers clear advantages over current nanogel or enzyme immobilization techniques.

## 2. Materials and Methods

### 2.1. Materials

All chemicals and reagents were utilized as received, without any additional purification. Methacrylated 2,2,6,6-tetramethyl-4-piperidyl (PMA, purity > 99%) was purchased from TCI Chemicals Pvt. Ltd., Tokyo, Japan. Glycidyl methacrylate (GMA, ≥97%) and poly(ethylene glycol) methyl ether 4-cyano-4-[(dodecylsulfanylthiocarbonyl)sulfanyl] pentanoate (PEG macro-CTA; Mn = 8–10 kDa) were procured from Sigma-Aldrich (Saint Louis, MO, USA). Azobisisobutyronitrile (AIBN, ≥98%) was also obtained from Sigma-Aldrich. Oxidized low-density lipoprotein (Ox-LDL) and Oil Red O solution (0.5% in isopropanol) were supplied by Sigma-Aldrich. Nunc™ MicroWell™ 96-well plates were sourced from Thermo Fisher Scientific (Altrincham, UK). Additional materials, including the Total Antioxidant Capacity Assay Kit, Cell Counting Kit-8 (CCK-8), Dulbecco’s Phosphate Buffered Saline (PBS, cell culture grade), and Dulbecco’s Modified Eagle’s Medium (DMEM), were all acquired from Sigma-Aldrich.

### 2.2. Instrumentation

#### 2.2.1. FTIR Spectroscopy

Fourier-transform infrared (FTIR) spectra were obtained for freeze-dried solid RAFT copolymer samples at ambient temperature. Each spectrum was recorded with an accumulation of 60 scans using a Nicolet FTIR spectrometer equipped with a diamond ATR accessory (Thermo Fisher Scientific, Waltham, MA, USA). Data acquisition and processing were performed using Spectrum software (PerkinElmer, Waltham, MA, USA; https://www.perkinelmer.com).

#### 2.2.2. Dynamic Light Scattering (DLS)

Particle size distribution and surface charge (zeta potential) of the nanoparticles were analyzed via Dynamic Light Scattering (DLS) using a Malvern Zetasizer Nano-ZS system (Malvern Panalytical Ltd., Malvern, UK). Measurements were taken in backscatter mode at a detection angle of 173°, with samples prepared at a concentration of 1.2 mg/mL. Each dataset comprised 16 scans and was repeated three times for consistency. Analyses were conducted at room temperature in deionized water [16]. 

#### 2.2.3. Size-Exclusion Chromatography

A 300 mm × 7.5 mm Agilent column was used for SEC analysis. Prior to each run, the column was equilibrated for 30 min. A 200 μL sample was injected, and the analysis was performed at 37 °C using a refractive index (RI) detector. The mobile phase consisted of N,N-dimethylformamide (DMF) containing 40 mM LiCl, flowing at 0.25 mL/min. Calibration was achieved with polyethylene glycol (PEG) standards covering a molecular weight range of 650 ≤ M/Da ≤ 9.42 × 10^5^. The chromatographic data were processed with TriSec software. Data analysis was performed using TriSEC software (https://www.wyatt.com).

#### 2.2.4. Transmission Electron Microscopy (TEM)

Nanogels suspensions (2.5 mg/mL) were deposited onto glow-discharged, lacy carbon-coated 300-mesh copper grids. Imaging was performed using a transmission electron microscope operating at an accelerating voltage of 200 keV.

#### 2.2.5. Spark^®^ Multimode Microplate Reader Tecan

A Spark^®^ multimode 96-well microplate reader (Tecan) (Tecan Group Ltd., Männedorf, Switzerland) was employed to record UV–visible absorption spectra for various bioassays [17].

### 2.3. Synthesis of RAFT Block Copolymer

**Figure 2:**
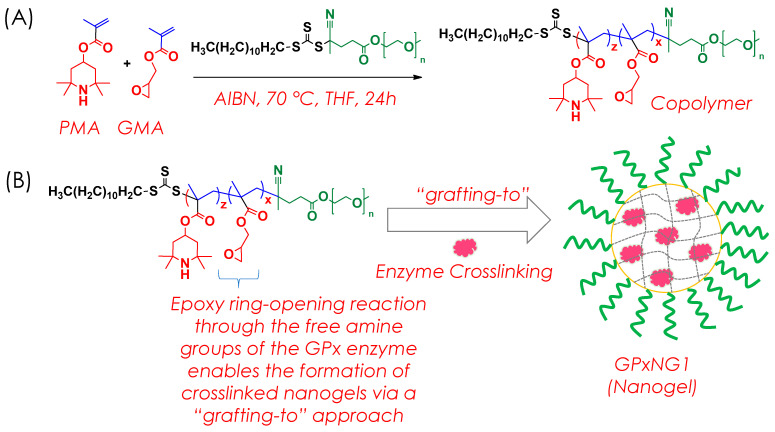
Schematic of copolymer synthesis and nanogel formation: (**A**) RAFT polymerization of the PEG macro-CTA with PMA and GMA; (**B**) epoxy–amine “grafting-to” reaction forming GPx-crosslinked nanogels.

The RAFT copolymer was synthesized through a reversible addition–fragmentation chain-transfer (RAFT) polymerization process (Figure 2A). Equimolar quantities of two monomeric units, methacrylated 2,2,6,6-tetramethyl-4-piperidyl (PMA, 0.25 mmol) and glycidyl methacrylate (GMA, 0.25 mmol), were polymerized in the presence of PEG macro-CTA (0.005 mmol) and azobisisobutyronitrile (AIBN, 0.0025 mmol) using tetrahydrofuran (THF) as the solvent. The reaction mixture was prepared in a 20 mL vial equipped with a magnetic stir bar and purged with nitrogen gas for 30 min at ambient temperature to ensure an oxygen-free environment.

After degassing, the sealed vial was transferred to a preheated polymerization block maintained at 65–70 °C and stirred continuously for 24 h. The polymerization was terminated by cooling the reaction mixture in an ice bath and exposing it to air. To eliminate any residual monomers and chain transfer agent (CTA), the crude product was precipitated into an excess of n-hexane, yielding a turbid suspension. The mixture was centrifuged at 3000 rpm for 10 min, and the supernatant layer of n-hexane was decanted. This purification cycle was repeated 6–8 times with fresh n-hexane. The molecular weight (Mw) of the purified copolymer was characterized using size-exclusion chromatography (SEC).

### 2.4. Synthesis of GPx Enzyme Conjugated Nanogels

For the preparation of GPx-conjugated nanogels, the RAFT copolymer and glutathione peroxidase (GPx) enzyme were combined in defined molar ratios (Figure 2B). The polymer concentration was maintained at 150 μM, while the enzyme concentration was varied between 10 and 50 μM. Both components were dissolved in 3 mL of phosphate-buffered saline (PBS, pH 7.2) to obtain a homogeneous solution. The resulting mixture was stirred continuously at 37 °C for 2–4 h to facilitate covalent conjugation through crosslinking reactions between the copolymer’s functional groups and the free amine sites of the GPx enzyme. The detailed feed ratios of copolymer to enzyme used in the synthesis are presented in Table 1.

After completion of the reaction, the nanogel dispersion was subjected to purification via centrifugation in deionized (DI) water to remove unreacted materials and other impurities. The collected nanogels were subsequently dialyzed against water using a semi-permeable membrane to eliminate residual low-molecular-weight species and excess polymer chains. This dialysis step allowed small molecules to diffuse out while retaining the nanogel structures within the membrane.

The purified and dialyzed GPx-conjugated nanogels were subsequently characterized using standard analytical techniques to assess their physicochemical and functional properties.

### 2.5. Nanogels Stability Test

The storage stability of the synthesized nanogels was evaluated over a period of one month at 4 °C. After the storage period, we analyzed the hydrodynamic size of the nanogels in both phosphate-buffered saline (PBS) and Milli-Q water at 25 °C. Dynamic Light Scattering (DLS) measurements were performed using a Malvern Zetasizer Nano-ZS instrument equipped with a backscatter detector operating at an angle of 173° (Malvern Panalytical Ltd., Malvern, UK). For each sample, three independent measurements were recorded, with each run consisting of 15–16 sub-scan cycles to ensure reproducibility and accuracy of the results. All experiments were conducted in triplicate to confirm data consistency.

### 2.6. Rheological Characterization

The viscoelastic properties of the nanogels were examined using a Hybrid Rheometer HR-2 (TA Instruments, New Castle, DE, USA) equipped with parallel plate geometry (20 mm diameter). All measurements were performed under swollen conditions at room temperature, maintaining a constant gap of 50 μm between the plates. We evaluated the viscoelastic behavior using an angular frequency sweep over a strain range of 0.1% to 100%. The complex viscosity was measured under the same conditions.

### 2.7. Nanogels Injectability Test

The injectability of the nanogels was evaluated in phosphate-buffered saline (PBS) at room temperature. Owing to the presence of nitroxide radicals within the polymer backbone, the nanogels exhibited a distinct orange color, allowing easy visual observation. For the injectability assessment, the nanogel sample was loaded into a 1 mL syringe fitted with an HSW HENKE-JECT^®^ (Henke-Sass, Wolf GmbH, Tuttlingen, Germany) disposable needle (19 G × 1″). A continuous shear force was applied to extrude the nanogel through the needle, and the injection process was visually recorded to confirm smooth flow behavior.

### 2.8. Cell Viability Study

The cytotoxicity of the three different nanogel formulations was evaluated using the Cell Counting Kit-8 (CCK-8, Sigma-Aldrich, Gillingham, UK). RAW 264.7 macrophage cells were cultured in T-75 flasks containing Dulbecco’s Modified Eagle Medium (DMEM) supplemented with 10% fetal bovine serum (FBS) and 1% penicillin. Cells between passages 6 and 8 were used for all experiments. The RAW 264.7 cell line (ATCC^®^ TIB-71™, Lot No.: 23A1234) was obtained from ATCC and supplied pre-authenticated by the vendor. The cells were confirmed to be mycoplasma-free using the supplier’s certificate of analysis, and no additional modifications were made to the cell line.

For the assay, healthy RAW 264.7 cells were seeded into 96-well plates at a density of 4.5 × 10^3^ cells per well in 500 μL of complete DMEM (10% FBS and 1% penicillin) and incubated for 24 h at 37 °C in a humidified atmosphere containing 5% CO_2_. Following incubation, the cells were treated with varying concentrations of nanogels and further incubated for 24 and 48 h to evaluate time-dependent cytotoxic effects.

After each incubation period, cell viability was determined by measuring the absorbance at 450 nm using a microplate reader after background correction. Phosphate-buffered saline (PBS) was used as the positive control, whereas Triton X-100 served as the negative control. All experiments were carried out in triplicate to ensure data reliability and reproducibility.

### 2.9. MDA-Lipid Oxidation Assay

The malondialdehyde (MDA) or thiobarbituric acid reactive substance (TBARS) assay was performed according to the manufacturer’s protocol provided with the Lipid Peroxidation (MDA) Assay Kit (Sigma-Aldrich, UK). In brief, copper-oxidized low-density lipoprotein (Ox-LDL) samples were prepared by adding 100 μL of phosphate-buffered saline (PBS) and 400 μL of 50 mM H_2_SO_4_ into a 2 mL Eppendorf tube. Subsequently, 150 μL of phosphotungstic acid was introduced, and the mixture was vortexed for 5 min. The samples and standards were then incubated at room temperature for 15 min, followed by centrifugation at 10,000× *g* for 10 min.

For LDL oxidation, 10 μM Cu^2+^ was added, and the reaction mixture was incubated at 37 °C for 4 h. After oxidation, excess copper ions were removed by dialysis, both with and without treatment using nanogels (NGs) or copolymers. The supernatant layer was collected and mixed with 10 μL of butylated hydroxytoluene (BHT) dissolved in 100 μL of deionized water and then brought to a final volume of 200 μL. The samples were subsequently heated at 100 °C for 45 min. Following incubation, 600 μL of thiobarbituric acid (TBA) solution was combined with 200 μL of the analyte mixture, and the absorbance was measured at 532 nm using a UV—visible spectrophotometer.

### 2.10. Total Antioxidant Capacity (TAC) Assay

The total antioxidant capacity (TAC) of the polymeric nanogels (RNONG) was evaluated using the Total Antioxidant Capacity Assay Kit (Sigma-Aldrich, MAK187) following the manufacturer’s instructions. The Cu^2+^ working solution was prepared by diluting the Cu^2+^ reagent with 49 parts of assay diluent. A Trolox standard stock solution (1 mM) was obtained by dissolving Trolox in 20 μL of dimethyl sulfoxide (DMSO) and diluting it with 980 μL of deionized (DI) water. A calibration curve was generated by adding the Cu^2+^ working solution to serially diluted Trolox standards.

For sample analysis, nanogel (NG) suspensions were prepared in DI water, and their absorbance was measured at 570 nm using a microplate reader. Each measurement was performed in triplicate to ensure reproducibility.. SBPG copolymer samples mixed with assay buffer served as controls.

The total antioxidant concentration of each sample was determined using the Trolox standard curve. The antioxidant capacity (expressed as nmol Trolox equivalents per μL of sample) was calculated using the following Equation (1):

(1) $$Antioxidant\;concentration=\frac{Sa}{Sv}\;$$

where $S_{a}$ represents the amount of Trolox equivalent (nmol) obtained from the standard curve, and $S_{v}$ denotes the sample volume (μL) used per well.

### 2.11. Activity, Stability, and Reusability Study

The catalytic activity of native glutathione peroxidase (GPx) and GPx-loaded nanogels was determined spectrophotometrically using the coupled enzyme assay with glutathione reductase. The decrease in NADPH absorbance at 340 nm was monitored at 25 °C. The reaction mixture consisted of phosphate buffer, reduced glutathione, NADPH, and hydrogen peroxide. Enzyme activity was expressed as nanomoles of NADPH oxidized per minute per milligram of protein.

To evaluate stability, samples were stored at 4 °C, and residual activity was measured at predetermined intervals. Additionally, the activity of freeze-dried samples was assessed under identical conditions. Reusability of GPx-loaded nanogels was examined by repeated catalytic cycles with intermediate washing. All experiments were performed in triplicate to ensure reproducibility and statistical reliability.

Reusability was evaluated over five consecutive catalytic cycles. After each cycle, the GPx-loaded nanogels were collected by gentle centrifugation, washed twice with phosphate buffer (pH 7.4), and reintroduced into a fresh reaction mixture under the same assay conditions. Enzymatic activity was measured at the start of each cycle to assess performance retention.

## 3. Results and Discussion

Nanogels are soft, biocompatible, and swellable polymeric nanoparticles that serve as versatile carriers for enzyme and drug delivery applications [18]. In this study, we report the design and fabrication of a novel glutathione peroxidase (GPx)-loaded nanogel system to enhance the enzyme’s catalytic stability, storage life, and reusability [19]. Native GPx enzyme is known to be unstable during long-term storage and undergoes activity loss after freeze–thaw cycles. To address this limitation, GPx enzyme was covalently grafted onto a reactive copolymer matrix through epoxy amine coupling, forming stable and soft nanogels that protect and preserve the enzyme’s redox activity.

**Figure 3:**
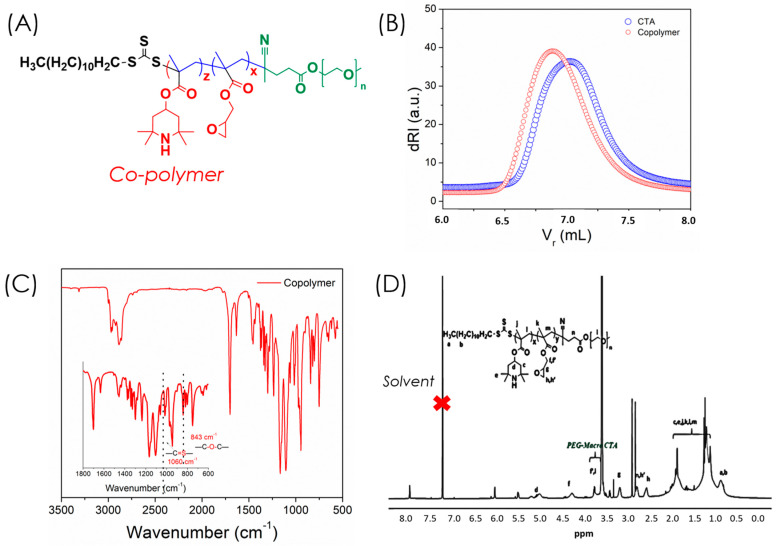
Structural characterization of the RAFT-synthesized copolymer: (**A**) copolymer structure with epoxy groups and PEG macro-CTA; (**B**) SEC traces of the macro-CTA and copolymer; (**C**) ATR-FTIR confirming epoxy and RAFT end-group signals; (**D**) ^1^H NMR spectrum in D_2_O.

An amphiphilic copolymer was synthesized via reversible addition–fragmentation chain transfer (RAFT) polymerization using methacrylated 2,2,6,6-tetramethyl-4-piperidyl (PMA, M1) and glycidyl methacrylate (GMA, M2) at a 50:50 molar ratio with PEG macro-CTA as the chain transfer agent (Figure 1) [20]. RAFT polymerization enabled precise control of the polymer chain length and molecular weight distribution (Figure 3A). The obtained copolymer exhibited a molecular weight (Mₚ, SEC) of 11,200 Da (Ð = 1.13), confirming successful polymerization compared to the PEG macro-CTA (8130 Da) (Figure 3B). Characteristic peaks in FTIR and ^1^H NMR spectra (Figure 3C,D) verified the successful formation of the copolymer and incorporation of epoxy groups. The epoxy vibrations observed at 926 and 843 cm^−1^ confirmed the presence of epoxide moieties, while the band at 1060 cm^−1^ corresponding to the trithiocarbonate (C=S) unit indicated the living nature of RAFT polymerization [21]. Only FTIR, ^1^H NMR, SEC, and UV–Vis analyses were performed in this work to confirm copolymer formation and evaluate nanogel function. Advanced structural techniques such as ^13^C NMR, XPS, SEM, and TEM were not included, as the focus was on establishing the epoxy–amine ‘grafting-to’ strategy and its functional performance; these will be incorporated in future studies for more comprehensive structural validation.

**Figure 4:**
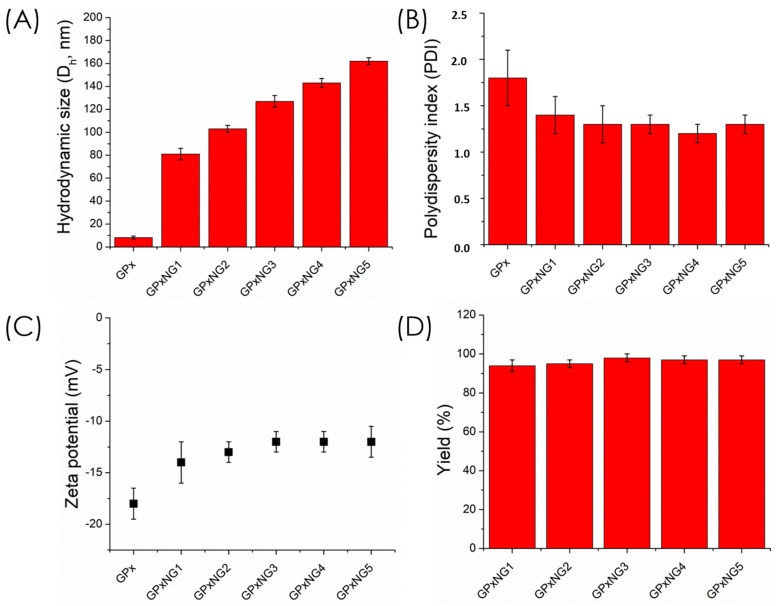
(**A**) Hydrodynamic size of GPx enzyme-grafted nanogels measured in aqueous medium using DLS; (**B**) polydispersity index (PDI) of various nanogels; (**C**) zeta potential of GPx-grafted nanogels; (**D**) overall yield (%) of enzyme GPx-grafted nanogels. Data are shown as mean ± SD (*n* = 3).

GPx-loaded nanogels (GPxNGs) were fabricated using an emulsion-free aqueous “grafting-to” approach, where epoxy groups reacted with the enzyme’s surface amine residues to form covalent crosslinks. Dynamic light scattering (DLS) analysis showed that the hydrodynamic diameter of GPxNGs increased with higher enzyme loading, indicating successful encapsulation (Figure 4A). Figure 4A shows that the particle size increased progressively from GPxNG1 to GPxNG5 as more GPx was incorporated, reflecting enhanced crosslinking between epoxy groups and enzyme amines. Figure 4B illustrates a reduction in PDI compared to the free enzyme, indicating the formation of uniform nanogel populations with improved colloidal homogeneity. As shown in Figure 4C, the zeta potential shifted toward less negative values after GPx conjugation, which further confirms successful attachment of the enzyme to the polymer network. The polydispersity index (PDI) values of GPxNGs were lower than those of native GPx enzyme solutions, reflecting improved size uniformity (Figure 4B). The zeta potential of GPxNGs was approximately −12 mV, slightly more positive than the copolymer alone, consistent with enzyme conjugation [22]. After purification by dialysis, yields of 96–98% were achieved (Figure 4C,D). Although direct spectroscopic techniques such as XPS or SDS–PAGE were not performed, the formation of GPx–polymer nanogels is strongly supported by indirect evidence, including increased hydrodynamic size, altered zeta potential, high purification yields after dialysis, gel-like rheological behavior, and markedly enhanced enzymatic stability. These combined physicochemical and functional results confirm successful nanogel formation.

**Figure 5:**
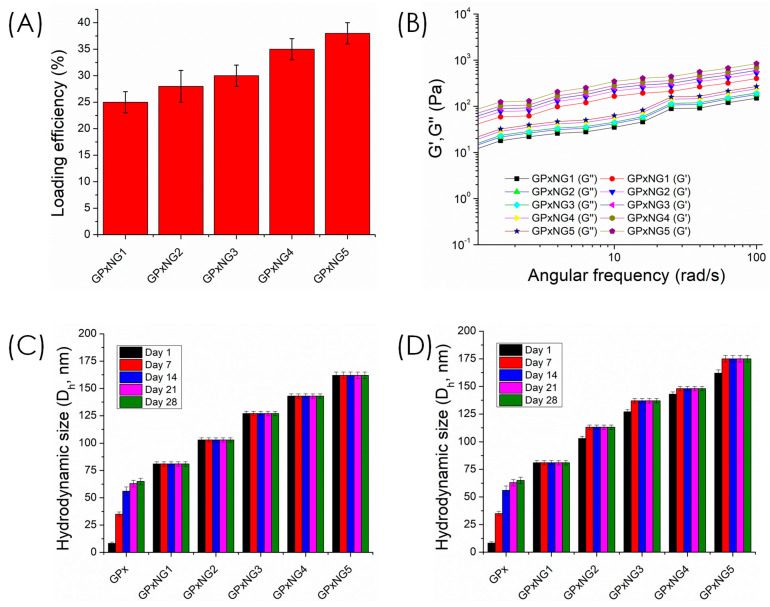
(**A**) Loading efficiency of various GPx-loaded nanogels; (**B**) rheological behavior of the nanogels, where G′ represents the storage modulus, and G″ represents the loss modulus as a function of angular frequency (rad/s); (**C**,**D**) storage stability of different nanogels compared with pristine GPx enzyme in Milli-Q water and PBS buffer media, respectively.

**Figure 6:**
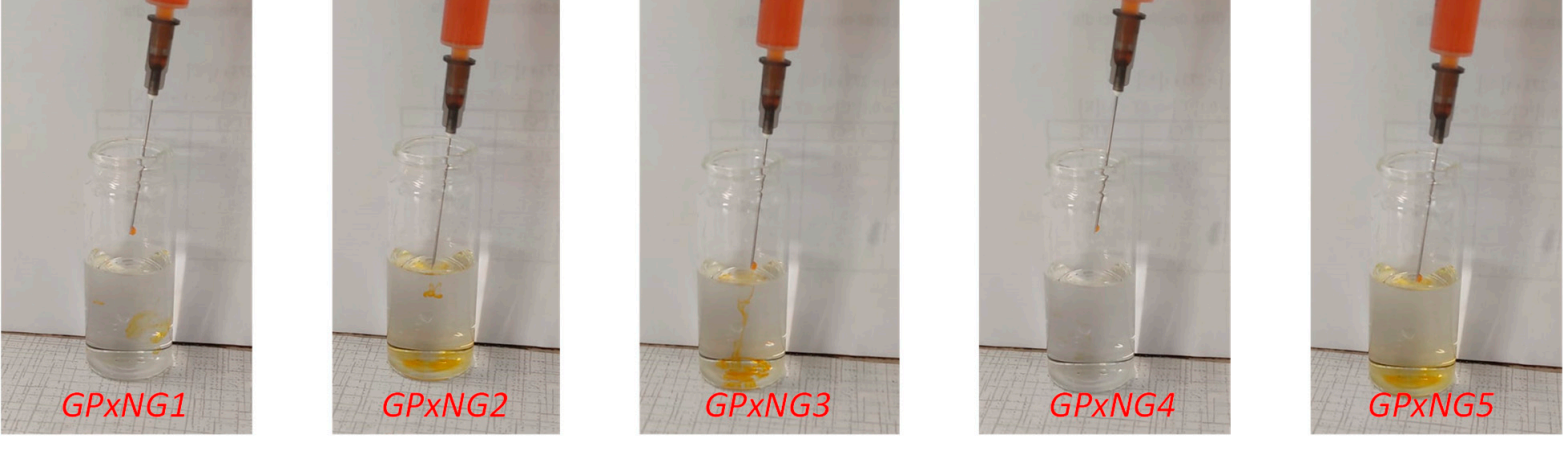
Shear-dependent rheological behavior and injectability of various GPx enzyme-loaded nanogels.

Loading efficiency of various GPx-loaded nanogels was reported in Figure 5A. The loading efficiency increased from GPxNG1 to GPxNG5 because higher enzyme concentrations provided more accessible amine groups for epoxy–amine coupling, resulting in more efficient crosslinking and greater incorporation of GPx into the nanogel network. Rheological analysis demonstrated that all GPxNGs exhibited storage modulus (G′) values higher than loss modulus (G″), indicative of their viscoelastic and gel-like nature (Figure 5B). The colloidal stability of GPxNGs was examined in both deionized water and PBS buffer (Figure 5C,D). The nanogels remained stable for at least four weeks at 4 °C without precipitation or turbidity, confirming their robust aqueous stability. Furthermore, shear-dependent viscosity tests revealed shear-thinning behavior, ensuring that the nanogels are easily injectable through standard medical needles without structural disruption (Figure 6). Such characteristics are essential for biomedical and drug delivery applications [23,24].

**Figure 7:**
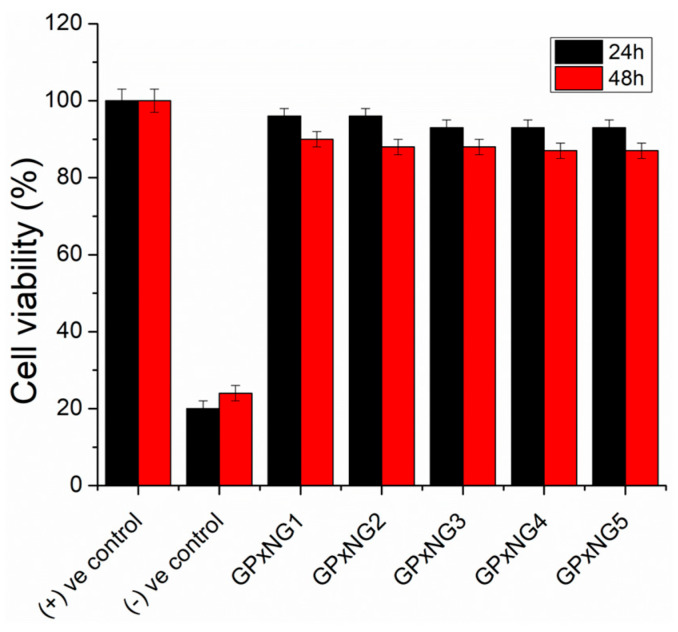
Cytocompatibility of PEGylated GPxNGs evaluated in RAW 264.7 macrophage cell cultures at 37 °C for 24 and 48 h using DMEM medium.

To assess cytocompatibility, PEGylated GPxNGs were tested in RAW264.7 macrophage cell cultures. The PEG shell surrounding the enzyme core is expected to enhance biocompatibility and minimize non-specific interactions. Cells cultured in the presence of GPxNGs exhibited normal morphology and proliferation (Figure 7). CCK-8 viability assays confirmed that GPxNGs were non-toxic, demonstrating excellent cytocompatibility suitable for biological use [25,26].

**Figure 8:**
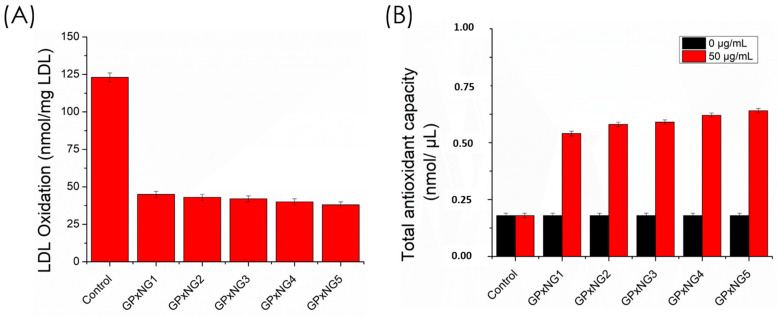
(**A**) MDA assay demonstrating the LDL oxidation quenching effect of various GPx-loaded samples compared with buffer media as the control; (**B**) total antioxidant capacity (TAC) assay of GPx-loaded nanogels evaluated at two different concentration ranges.

The antioxidant performance of GPxNGs was compared to native GPx enzyme using lipid peroxidation and free radical scavenging assays. Oxidative stress induces lipid peroxidation of polyunsaturated fatty acids (PUFAs), generating lipid hydroperoxides (LOOH) and peroxyl radicals (LOO•), which damage cell membranes. GPx enzyme catalyzes the reduction of LOOH to less reactive products, thus preventing oxidative injury. The malondialdehyde–thiobarbituric acid reactive substance (MDA–TBARS) assay revealed that GPxNGs exhibited stronger ROS scavenging activity than native GPx enzyme, maintaining catalytic efficiency even after prolonged storage (Figure 8A,B). This enhanced performance is attributed to the protective nanogel matrix, which shields GPx enzyme from denaturation and preserves its active conformation [27,28,29].

The LDL oxidation quenching effect showed only minor variation among the GPx enzyme nanogels because LDL oxidation is a multi-step lipid and protein oxidation process that becomes saturated once sufficient GPx activity is present. As a result, additional GPx incorporation does not proportionally increase inhibition. In contrast, the TAC assay directly measures the total reducing capacity of the nanogels, which increases linearly with higher GPx loading. Therefore, the TAC values show a gradual enhancement, whereas the LDL oxidation assay exhibits smaller differences between samples.

We further examined the role of GPxNGs in mitigating oxidative LDL modification, a key event in atherosclerosis progression. Transition metal ions such as Cu^2+^ and Fe^2+^ catalyze the breakdown of LOOH into reactive radicals (Equations (2) and (3)), accelerating LDL oxidation. GPxNGs effectively suppressed this process, demonstrating superior antioxidant capacity compared to the free enzyme (Figure 8). The total antioxidant capacity (TAC) assay using the Trolox standard confirmed the robust radical scavenging potential of GPxNGs [30,31].

(2) $$LOOH+{M(II)}^{+}\to {LOO}^{.}+H^{+}+{M(I)}^{+}$$

(3) $${M(I)}^{+}+LOOH\to {LO}^{.}+{OH}^{-}+{M(II)}^{+}$$

**Figure 9:**
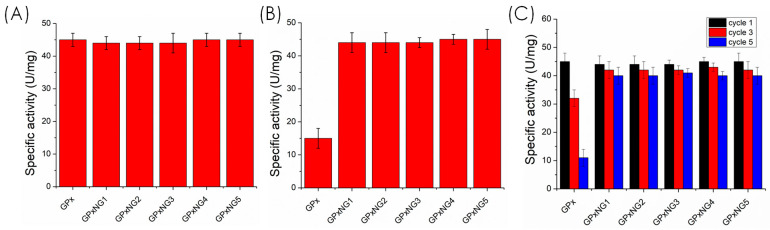
(**A**) Specific activity of free GPx enzyme compared with GPx-loaded nanogels; (**B**) comparison of specific activity between GPx enzyme and GPx-loaded nanogels after storage at 4 °C; (**C**) bar graph showing GPx enzyme and GPx-loaded nanogels activity across different freeze-dried samples (cycles 1, 3, and 5).

To validate the functional stability of the enzyme, the catalytic activity of stored and freeze-dried GPxNGs was compared with that of native GPx. The nanogels retained significantly higher activity after multiple freeze–thaw cycles and four weeks of storage, whereas the free enzyme showed marked activity loss (Figure 9). This protective effect arises from the confinement of the GPx enzyme within the flexible polymeric network, which minimizes structural unfolding and aggregation. Moreover, GPxNGs maintained catalytic efficiency after repeated use, confirming their reusability [32,33,34]. While the formation of GPx–polymer conjugates was supported by DLS, zeta potential changes, high purification yields, and preserved enzymatic function, direct spectroscopic confirmation (e.g., XPS, SDS–PAGE) was not performed in this study. These mechanistic analyses will be included in future work to further validate the covalent epoxy–amine “grafting-to” binding mechanism. This study focused specifically on the covalent epoxy–amine “grafting-to” strategy for GPx nanogel formation; therefore, non-PEGylated, physically adsorbed, or blank nanogel controls were not included. These additional formulations will be examined in future studies to provide a broader comparative evaluation.

## 4. Conclusions

This work presents a PEGylated glutathione peroxidase nanogel engineered through a mild “grafting-to” epoxy–amine coupling strategy to enhance enzyme stability and antioxidant performance. The RAFT-derived PMA-co-GMA copolymer enabled efficient GPx conjugation, yielding uniform, soft, and injectable nanogels with excellent colloidal stability and cytocompatibility. Notably, GPx nanogels retained ~95% of their catalytic activity even after five freeze–thaw cycles, demonstrating exceptional structural protection compared to the rapid activity loss observed for the free enzyme. The nanogels also showed superior ROS-scavenging efficiency and strong inhibition of lipid peroxidation and LDL oxidation. Overall, these findings establish GPx nanogels as a robust and biocompatible platform for stabilizing redox enzymes and advancing antioxidant therapeutic strategies for oxidative stress-related diseases.

## Figures and Tables

**Table 1:** The optimization of nanogel formation was carried out by employing the free amine groups of the GPx enzyme as both crosslinking and conjugation sites at a controlled temperature of 37 °C. Throughout the optimization process, the copolymer concentration was maintained constant, while the enzyme concentration was systematically varied. All other reaction parameters were kept unchanged to ensure consistency in evaluating the effect of enzyme concentration on nanogel synthesis.

Sample No.	Polymer (μM)	Enzyme (μM)	Buffer (pH)	Temperature (°C)
**GPxNG1**	150	10	7.2	37
**GPxNG2**	150	20	7.2	37
**GPxNG3**	150	30	7.2	37
**GPxNG4**	150	40	7.2	37
**GPxNG5**	150	50	7.2	37

## Data Availability

The original contributions presented in this study are included in the article. Further inquiries can be directed to the corresponding author.
